# LSM12 facilitates the progression of colorectal cancer by activating the WNT/CTNNB1 signaling pathway

**DOI:** 10.32604/or.2022.028225

**Published:** 2023-02-09

**Authors:** YAN ZHUANG, CHUNLAN NING, PENGFEI LIU, YANPENG ZHAO, YUE LI, ZHENCHI MA, LULING SHAN, YINGZHE PIAO, PENG ZHAO, XUN JIN

**Affiliations:** 1 Department of Colorectal Oncology, Tianjin Medical University Cancer Institute & Hospital, National Clinical Research Center for Cancer, Tianjin’s Clinical Research Center for Cancer, Key Laboratory of Cancer Prevention and Therapy, Tianjin, 300060, China; 2 Department of Biochemistry and Molecular Biology, Tianjin Medical University Cancer Institute and Hospital, National Clinical Research Center for Cancer, Tianjin’s Clinical Research Center for Cancer, Key Laboratory of Cancer Prevention and Therapy, Tianjin, 300060, China; 3 Tianjin Medical University, Tianjin, 300070, China; 4 Department of Oncology, Tianjin Academy of Traditional Chinese Medicine Affiliated Hospital, Tianjin, 300120, China; 5 Tianjin Marvel Medical Laboratory, Tianjin Marvelbio Technology Co., Ltd., Tianjin, 300381, China; 6 Department of Gastro Colorectal Oncology, Tianjin Cancer Hospital Airport Hospital, Tianjin, 300304, China; 7 Department of Neuro-Oncology and Neurosurgery, Tianjin Medical University Cancer Institute & Hospital, National Clinical Research Center for Cancer, Key Laboratory of Cancer Prevention and Therapy Tianjin, Tianjin’s Clinical Research Center for Cancer, Tianjin, 300060, China

**Keywords:** CRC, LSM12, WNT, CTNNB1, Apotosis

## Abstract

Aberrant activation of the WNT signaling pathway is a joint event in colorectal cancer (CRC), but the molecular mechanism is still unclear. Recently, RNA-splicing factor LSM12 (like-Sm protein 12) is highly expressed in CRC tissues. This study aimed to verify whether LSM12 is involved in regulating CRC progression via regulating the WNT signaling pathway. Here, we found that LSM12 is highly expressed in CRC patient-derived tissues and cells. LSM12 is involved in the proliferation, invasion, and apoptosis of CRC cells, similar to the function of WNT signaling in CRC. Furthermore, protein interaction simulation and biochemical experiments proved that LSM12 directly binds to CTNNB1 (also known as β-Catenin) and regulates its protein stability to affect the CTTNB1-LEF1-TCF1 transcriptional complex formation and the associated WNT downstream signaling pathway. LSM12 depletion in CRC cells decreased the *in vivo* tumor growth through repression of cancer cell growth and acceleration of cancer cell apoptosis. Taken together, we suggest that the high expression of LSM12 is a novel factor leading to aberrant WNT signaling activation, and that strategies targeting this molecular mechanism may contribute to developing a new therapeutic method for CRC.

## Introduction

Colorectal cancer (CRC) is the 3^rd^ most common malignant tumor and the 2^nd^ leading cause of cancer-related death all over the world [1]. Interestingly, 96% of CRC patients have mutations in WNT (Wingless-type)/CTNNB1 (β-Catenin) signaling pathway components such as APC (adenomatous polyposis coli) (79%), CTNNB1 (8%), RNF43 (ring Finger protein 43) (9%) [2]. In WNT/CTNNB1 signaling pathway, the WNT ligand binds to Fz (Frizzled)/LRP (low-density lipoprotein receptor-related protein) receptor complex. It accelerates CTNNB1 accumulation in the cytoplasm and nucleus by blocking the GSK3 (glycogen synthase kinase 3)/APC/Axin complex-mediated CTNNB1 protein degradation by recruiting Dsh (Dishevelled). And Nuclear CTNNB1 can bind to TCF (T cell-specific transcription factor)/LEF (lymphoid enhancer factor) to form a transcriptional regulatory complex (also termed WNT enhanceosome) to transcript their target genes such as cMYC, c-JUN, WISP1 (Wingless-type inducible signaling pathway protein-1) and PPARD (peroxisome-proliferator-activated receptor D), which is involved in the regulation of cell survival, proliferation, differentiation, migration, and apoptosis [3]. Therefore, further elucidation of the regulatory mechanism of the WNT signaling pathway in CRC has a great significance in developing the strategy for CRC diagnosis and therapy.

Although high-frequency APC mutations in CRC prevent the degradation of CTNNB1 protein, stable CTNNB1 protein is not required to enter the nucleus to perform transcriptional regulatory functions. In fact, CTNNB1 usually plays an important role in stabilizing the polarity of epithelial cells, including CRC cells, by binding to E-cadherin on cell membranes [4]. These mechanisms suggest that activation of WNT signaling in CRC requires proteins. It regulates the distribution of CTNNB1 proteins into the nucleus and the formation of transcriptional regulatory complexes of CTNNB1 with TCF/LEF. A recent study discovered that RNA-splicing factor LSM12 (Like-Sm protein 12) played a nucleocytoplasmic transport for sustaining the RAN gradient between the cytoplasm and nucleus [5], and was aberrantly overexpressed in CRC tissues when relative to adjacent normal tissues [6]. This evidence suggests that LSM12 may act as a nuclear localization regulator of CTNNB1 to activate WNT signaling for CRC malignant development abnormally. Thus, this study was implemented to verify whether LSM12 is involved in the regulation of CRC progression via regulating the WNT signaling pathway. The current work might provide innovative WNT signal targets and exact molecular mechanisms for CRC target treatment.

## Materials and Methods

### Human specimens

This study collected 30 pairs of clinical samples (including tumor tissue specimens and adjacent normal tissue specimens) from CRC cases. These patients were diagnosed with CRC in our hospital from April 2019 to September 2021. All of these cases have signed written informed consent before surgery. This work has been ratified by the Ethics Committee.

### Cell lines and culture

Human normal colorectal mucosal cell line (FHC) and CRC cell lines (SW620, SW480, HCT-116, Caco-2, LS174T, and HT-29) were purchased from the Shanghai JINyuan Biotechnology Co., Ltd. (Shanghai, China). The integrity of all cell lines was authenticated by short tandem repeat (STR) before use. The cell lines were grown in dulbecco’s modified eagle medium (DMEM; Solarbio, Beijing, China) with 10% fetal bovine serum (FBS; Solarbio, Beijing, China) in a 5% CO_2_ incubator at 37°C.

### Cell transfection

shRNA targeting LSM12 (#1, #2 and #3) and scramble control (Scr.) (GeneChem, Shanghai, China) were utilized to treat SW480 and HCT116 cells. Briefly, SW480 and HCT116 cells were grown in 1 mL serum-free DMEM in 6-well plates (Lianshuo Biotechnology, Shanghai, China) with 1 × 10^6^ cells per well. Then SW480 and HCT116 cells were transfected by LSM12 shRNA and shScr. according to the directions of Lipofectamine 2000 (ThermoFisher Scientific, Shanghai, China). After transfection, SW480 and HCT116 cells were cultured by DMEM containing 10% FBS for 48 h. The transfection efficiency was researched by real-time quantitative reverse transcription polymerase chain reaction (qRT-PCR) and Western blot.

### Construction of stable LSM12 knockdown cell lines and doxycycline (DOX) treatment

The Tet-On-regulated system can adequately control the expression of genes with good safety to cells. In this study, the knockdown of LSM12 in SW480 and HCT-116 cells was implemented by using the Tet-On-shLSM12 system and induced by DOX (Yuanye Biotechnology, Shanghai, China). Briefly, shRNA targeting LSM12 was synthesized by GeneChem (Shanghai, China) and then cloned into pLKO-Tet-On (2  μg, cat: 21915, Yipu Biotechnology Co., Ltd., Wuhan, China) strictly in line with the directions. pLKO-Tet-On-shLSM12 was utilized to transfect 293T cells (ATCC, Rockville, MD, USA) via using Lipofectamine 2000 (ThermoFisher Scientific, Shanghai, China) to generate lentiviral particles. At 48 and 72 h post-transfection, the supernatant was collected and filtered by employing membrane filters (0.45 μm pore size, Millipore Bedford, MA, USA), followed by being centrifuged for 2 h at 70,000  ×  g and 4°C. SW480 and HCT-116 cells were grown in the petri dish (6 cm in diameter; Absin Biotechnology, Shanghai, China) with 3 mL of DMEM and 1 mL of the lentiviral supernatant. Two days later, puromycin (1.5 μg/mL, cat: 60210ES25, Yeasen Biotechnology, Shanghai, China) was added to select SW480 and HCT-116 cells stably infected with LSM12 shRNA. Thereafter, these stably infected SW480 and HCT-116 cells were treated by DOX (100 ng/mL, cat: ST039B, Beyotime, Shanghai, China) for 48 h (set as DOX+ group). Those without DOX treatment were used as control (named DOX− group).

### ICG001 treatment of cells

ICG001 is a transcriptional inhibitor of the CTNNB1/TCF complex, which is often used to suppress the WNT signaling pathway in CRC cells [7]. This study used ICG001 (10 μM, cat: SF6827, Beyotime, Shanghai, China) to treat SW480 and HCT-116 cells (set as ICG001+ group). SW480 and HCT-116 cells in the absence of ICG001 were considered as control (named ICG001− group).

### Cell counting kit-8 (CCK-8) assay

The viability of SW480 and HCT-116 cells was researched by CCK-8 assay. SW480 and HCT-116 cells were inoculated in 96-well plates (Lianshuo Biotechnology, Shanghai, China) and cultured with DMEM in the presence of 10% FBS and DOX (100 ng/mL) or not (1 × 10^6^ cells per well). After being cultured for 24, 48, 72 and 96 h at 37°C, 5% CO_2_, SW480 and HCT-116 cells of each group were treated with 10 μL CCK-8 solution (Fuheng Biotechnology, Shanghai, China) for 2 h at 37°C. A multiwell microplate reader (Multiskan MK3, Thermo Scientific, MA, USA) was utilized to detect the optical density (OD) value.

### Colony formation assay

The colony formation ability of SW480 and HCT-116 cells was detected by colony formation assay. SW480 and HCT-116 cells of each group were cultured into 6-well plates with the relevant medium (1 mL) at 37°C, 5% CO_2_ (1 × 10^5^ cells per well). The relevant medium in each well was changed at a 3-day interval. After 14 days of culture, cells were washed twice with phosphate buffered saline (PBS; Solarbio, Beijing, China). For cells attached on the bottom, they were fixed by 4% paraformaldehyde (Beyotime Biotechnology, Shanghai, China) for 10 min, and stained 0.1% crystal violet (Beyotime Biotechnology, Shanghai, China) for 10 min. At last, the number of clones in each group were counted under a microscope (Olympus, Tokyo, Japan).

### Transwell experiment

The invasion and migration capacities of SW480 and HCT-116 cells were explored by the Transwell experiment. Transwell chambers (Yanhui Biotechnology, Shanghai, China) precoated with (or without) Matrigel (Beyotime, Shanghai, China) were inserted into 24-well plates (Lianshuo Biotechnology, Shanghai, China). SW480 and HCT-116 cells (1 × 10^6^ cells/mL) in the serum-free DMEM medium (200 µL) were inoculated into the upper chambers. For the lower chambers, DMEM containing 10% FBS was filled. Cells were stored at 37°C under 5% CO_2_ for 24 h. The invasion or migration cells underwent 10 min fixation via 4% paraformaldehyde (Beyotime Biotechnology, Shanghai, China) and then 10 min staining via 0.1% crystal violet (Beyotime Biotechnology, Shanghai, China). The counting of invading or migrating cells were implemented under a microscope (Olympus, Tokyo, Japan). For each group, five non-overlapping fields were randomly selected to count invading or migrating cells.

### Flow cytometry

Flow cytometry was utilized to investigate the apoptosis of SW480 and HCT-116 cells. In brief, SW480 and HCT-116 cells were harvested after 48 h of culture by the relevant medium. Pre-cooled PBS (Solarbio, Beijing, China) was utilized to wash cells. After 5 min of centrifugation (300 × g, 4°C), cell apoptosis was monitored by recruiting an Annexin V/PI Apoptosis Detection Kit (YB40303ES20, YBio, Shanghai, China) by referring to the directions. Apoptosis was monitored by applying flow cytometry (Becton-Dickinson, Mountain View, CA, USA).

### TUNEL assay

TUNEL assay is an effective method to evaluate the apoptosis of cells. SW480 and HCT-116 cells of each group were cultured for 48 h in the relevant medium in 6-well plates (Lianshuo Biotechnology, Shanghai, China). The TUNEL assay was implemented according to the instructions of the TUNEL kit (C1090, Beyotime, Shanghai, China). Briefly, cells were immersed into 0.01% Triton X-100 (Beyotime, Shanghai, China) for 10 min. PBS (Solarbio, Beijing, China) was recruited to wash cells twice. TUNEL working solution (Beyotime, Shanghai, China) was then added to treat cells for 1 h in the dark. The 4′6-diamidino-2-phenylindole (DAPI, Beyotime, Shanghai, China) was utilized to stain cells for 5 min in the dark. Cells were then washed twice with PBS. TUNEL positive cell (red fluorescence) counts were executed under fluorescence microscopy (Olympus, Tokyo, Japan).

### Coimmunoprecipitation (co-IP) experiment

293T cells were used for exogenous co-IP experiments to verify the interaction between LSM12 and CTNNB1. Briefly, pcDNA3.1-LSM12-Flag+pcDNA3.1-CTNNB1-MYC and pcDNA3.1-Flag+pcDNA3.1-CTNNB1-MYC (GeneChem, Shanghai, China) were transfected into 293T cells in line with the directions. After 48 h of transfection, cells were treated by immunoprecipitation lysis buffer (Solarbio, Beijing, China). The cell lysates experienced immunoprecipitation by incubation with anti-flag (ab205606, Abcam, Shanghai, China) and anti-cMYC (ab71676, Abcam, Shanghai, China) antibodies. Western blot was executed to investigate the expression of precipitated proteins. For the endogenous co-IP experiment, SW480 cells per group were cultured for 48 h in the relevant medium. Protein A/G magnetic beads (Amyjet Scientific, Wuhan, China) were added into the cell lysate for 2 h incubation at 4°C. Through centrifugation, these beads were removed, and the supernatant was incubated with primary antibodies (anti-LSM12 [ab173291, Abcam, Shanghai, China], anti-CTNNB1 [ab32503, Abcam, Shanghai, China], anti-LEF1 [ab2324, Abcam, Shanghai, China], anti-TCF1 [ab188865, Abcam, Shanghai, China]) or IgG (ab172730, Abcam, Shanghai, China) for 12 h at 4°C. Then protein A/G beads were employed to treat the immunoprecipitation mixture samples for 4 h. After being collected, these beads were washed twice by cooled IP lysis buffer (Beyotime Biotechnology, Shanghai, China) to remove the nonspecifically bound proteins. The specifically bound proteins were qualified by Western blot after being eluted from the beads.

### Cycloheximide (CHX) treatment

CHX is a protein synthesis inhibitor, often used to determine the degradation process of protein [8]. In this study, CHX (Beyotime Biotechnology, Shanghai, China) was used to treat SW480 cells to monitor the influence of LSM12 on the stability of the CTNNB1 protein. SW480-Tet-On-shLSM12 cells of DOX+ and DOX− group was cultured into 6-well plates (Lianshuo Biotechnology, Shanghai, China) at a density of 1 × 10^6^ cells/mL per well. CHX (50 μg/mL) was contained in the relevant medium. Cells were maintained at 37°C, and 5% CO_2_ for 0, 2, 4 and 6 h, respectively. At each specific time point, cells were harvested to monitor the expression of LSM12 and CTNNB1 proteins via Western blot.

### In vivo xenograft experiments

This research purchased 10 Balb/C nude mice (6 weeks old) from the SJA Laboratory Animal Co., Ltd. (Hunan, China). These mice were divided into the DOX+ group (n = 5) and the DOX− group (n = 5). SW480-Tet-On-shLSM12 cells (1 × 10^7^ cells) suspended in PBS (Solarbio, Beijing, China) were injected subcutaneously into the back of mice. Mice of the two groups were maintained in a room with a 12 h day/night cycle. Food and water were freely available. Seven days post-injection, mice of the DOX+ group were injected intravenously with DOX (4 mg/kg; Yuanye Biotechnology, Shanghai, China) at a 3-day interval. The length and width of the tumor mass were monitored at 7-day intervals. Tumor volume was determined by width^2^ * length * 0.5. Until 35 days, mice were sacrificed by rapid cervical dislocation after deep anesthesia with 5% isoflurane (Yuyan Scientific Instruments, Shanghai, China). All the tumor mass was stripped and weighed. Animal experiments were implemented after being approved by the Animal Ethics Committee.

### Immunohistochemistry

The expression of LSM12 and Ki67 proteins in the tumor tissues of mice was detected by immunohistochemistry. After being fixed via employing 10% formaldehyde (Yuanmu Biotechnology, Shanghai, China), tumor tissues were embedded into paraffin (Hengfei Biotechnology, Shanghai, China) and then prepared into sections (4 μm). Before antigen retrieval, the sections underwent xylene (Xinyu Biotechnology, Shanghai, China) and gradient alcohol (Xinyu Biotechnology, Shanghai, China) treatment for dewaxing and dehydration. The removal of endogenous peroxidase was implemented by incubating with 3% H_2_O_2_ (Xinyu Biotechnology, Shanghai, China). Afterward, goat serum (Beyotime Biotechnology, Shanghai, China) was recruited to treat the sections for blockage. LSM12 antibody (1:100, ab173292, Abcam, Shanghai, China) and Ki67 antibody (1:100, ab15580, Abcam, Shanghai, China) were utilized to probe the sections overnight at 4°C. After this, the sections were subjected to secondary antibody (1:200, ab6721, Abcam, Shanghai, China) treatment for 15 min at 37°C. Diaminobenzidine (DAB; Solarbio, Beijing, China) staining and the following hematoxylin (Solarbio, Beijing, China) counterstaining of the sections were sequentially executed. After being washed by PBS (Solarbio, Beijing, China) and sealed in neutral resin (Solarbio, Beijing, China), the sections were observed under a microscope (Olympus, Tokyo, Japan).

### qRT-PCR

The expression of mRNAs was assessed by qRT-PCR. According to the directions, total RNA extraction from tissues and cells was implemented by employing the RNA extraction kit (Applied Biosystems, Foster City, CA, USA) in line with the directions. For each total RNA sample, a reverse transcription reaction was executed to synthesize cDNA templates by utilizing the PrimeScript RT Reagent Kit (Takara, Dalian, China) following the directions. Subsequently, PCR was executed via recruiting the SYBR Green PCR Master Mix (Applied Biosystems, Foster City, CA, USA) on the ABI 7500 Fast Real-Time PCR system (Applied Biosystems, Foster City, CA, USA). The conditions for PCR were: 60 s at 94°C firstly, and then 40 cycles (30 s at 92°C, 30 s at 56°C, and 30 s at 74°C). The primers were: LSM12, forward, 5′-CTGTTCTTCCCACCTCA-3′, reverse, 5′-GCACACTGGCTCTACAAA-3′. cMYC, forward, 5′-CCAGAGGAGGAACGAG-3′, reverse, 5′-CTTGGACGGACAGGAT-3′. c-JUN, forward, 5′-GCGGACCTTATGGCTACA-3′, reverse, 5′-TGATGTGCCCGTTGCT-3′. WISP1, forward, 5′-AGCACACGCTCCTATCA-3′, reverse, 5′-AAGCCCATCAGGACACT-3′. PPARD, forward, 5′-GGAGCCAGTACAACCCA-3′, reverse, 5′-ACACCAGCCCCTTCTCT-3′. β-actin, forward, 5′-GTGGGCCGCTCTAGGCACCA-3′, reverse, 5′-CGGTTGGCCTTAGGGTTCAGGGGGG-3′. β-actin was regarded as the control. The relative expression of target genes was determined by the 2^−ΔΔCt^ method.

### Western blot

The expression of proteins was evaluated by Western blot. Total protein was extracted from tissues and cells using RIPA Lysis Buffer (Beyotime, Shanghai, China) according to the directions. Total protein concentration was executed by employing the BCA kit (Beyotime, Shanghai, China) according to the directions. Sodium dodecyl sulfate-polyacrylamide gel electrophoresis was thereafter implemented for the separation of proteins. Followed by this, the proteins were transferred onto a polyvinylidene fluoride (PVDF; Mingyang Kehua Biotechnology, Beijing, China) membrane and then experienced 30 min blocking by 5% skimmed milk (Beyotime Biotechnology, Shanghai, China). Primary antibodies were dropped onto the PVDF membrane to probe proteins overnight at 4°C. The information of rabbit anti-primary antibodies were as follows: LSM12 (1:1000, ab173292, Abcam, Shanghai, China), caspase-3 (1:1000, ab44976, Abcam, Shanghai, China), Cleaved caspase-3 (1:1000, ab2302, Abcam, Shanghai, China), caspase-9 (1:1000, ab25758, Abcam, Shanghai, China), Cleaved caspase-9 (1:1000, ab2324, Abcam, Shanghai, China), cMYC (1:1000, ab152146, Abcam, Shanghai, China), c-JUN (1:1000, ab32137, Abcam, Shanghai, China), WISP1 (1:1000, ab263948, Abcam, Shanghai, China), PPARD (1:1000, ab8937, Abcam, Shanghai, China), CTNNB1 (1:1000, PAB1228, Amyjet Scientific, Wuhan, China), LEF1 (1:1000, ab74549, Abcam, Shanghai, China), TCF (1:1000, ab134275, Abcam, Shanghai, China) and β-actin (1:1000, ab8227, Abcam, Shanghai, China). The sections then experienced the 2 h treatment by goat anti-rabbit secondary antibody (1:2000, ab6721, Abcam, Shanghai, China) at room temperature. The development of protein blots was implemented by referring to the directions of the ECL kit (Beyotime, Shanghai, China). Image J software (NI Health, Bethesda, MD, USA) was adopted to qualify the protein blots. β-actin was considered as the control.

### Bioinformatics analysis

The expression data of LSM12 in tumor tissue specimens and adjacent normal tissue specimens was analyzed based on the TCGA COAD (The Cancer Genome Atlas Colon Adenocarcinoma) dataset and the TCGA-READ (The Cancer Genome Atlas Rectum Adenocarcinoma Cohort) dataset. These data were collected from Xena of the GEPIA2 website, and transcriptomic data TPM was employed. Centralized processing was performed on these data.

The platform GPS-Prot (gpsprot.org) was used to monitor an interaction protein network with LSM12 protein under a min confidence score cutoff of 0.63 [9].

### Statistical analysis

In this study, data (mean ± Standard Error of Mean [SEM]) was analyzed and made into histograms by recruiting the Graphpad Prism 5.0 software. Data comparison between two groups was executed by a two-tailed paired Student’s *t*-test, and a one-way analysis of variance was implemented for the data comparison in multiple groups. *p* < 0.05 was regarded as a statistically significant difference.

## Results

### High LSM12 expression level occurred in CRC cases and cells

**Figure 1:**
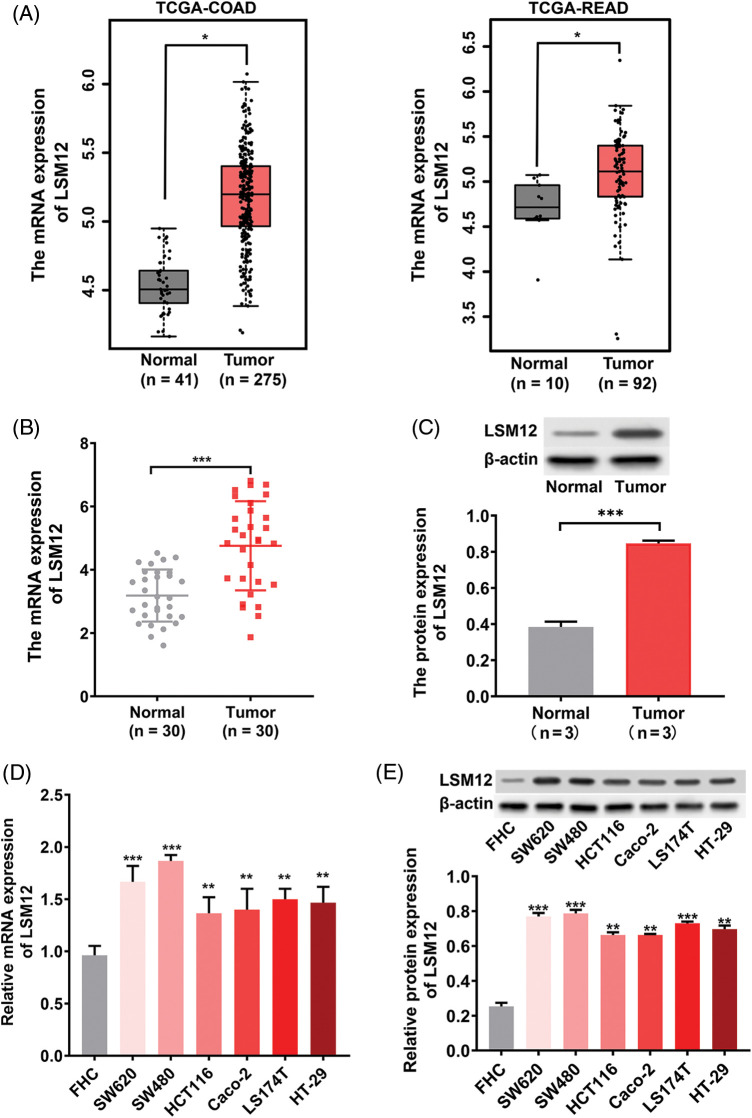
LSM12 expression in CRC specimens and cells. (A) The expression level of LSM12 mRNA in tumor (n = 275) and adjacent normal (n = 41) specimens from TCGA-COAD database, as well as in tumor (n = 92) and adjacent normal (n = 10) specimens from TCGA-READ database. *p < 0.05. (B) The expression level of LSM12 mRNA in CRC (n = 30) and adjacent normal (n = 30) specimens was revealed by qRT-PCR. ***p < 0.001. (C) The expression level of LSM12 protein in CRC (n = 3) and adjacent normal (n = 3) specimens was detected by Western blot. ***p < 0.001. Note: Wilcox test focused on the differences in medians, while the *t*-test focused on the differences in means. For the data in (C), three tissue samples were included for each group, so the mean was more representative than the median. Thus, the paired *t*-test was used for data comparison in (C). (D) Expression of LSM12 mRNA in CRC (SW620, SW480, HCT116, Caco-2, LS174T, HT-29) and normal (FHC) cell lines was determined by qRT-PCR. **p < 0.01. (E) The expression of LSM12 protein in CRC cell lines and normal cell lines was demonstrated by Western blot. **p < 0.01 or ***p < 0.001. All cell line-based experiments were independently conducted in triplicate technical repeats. Data shown are means ± SEM.

To evaluate the tumorigenic potential of LSM12 in CRC, we first analyzed the expression of LSM12 in the TCGA-COAD and TCGA-READ databases. We found that LSM12 was significantly overexpressed in tumor specimens compared with adjacent normal specimens (*p* < 0.05) in COAD patients and READ patients (Fig. 1A). This result was confirmed at mRNA and protein levels in our paired CRC clinical samples (*p* < 0.001) (Figs. 1B and 1C). Similarly, in comparison to human normal colorectal mucosal cell line (FHC), distinctly higher levels of LSM12 mRNA and protein occurred in CRC cell lines (SW620, SW480, HCT-116, Caco-2, LS174T, and HT-29) (*p* < 0.01 or *p* < 0.001) (Figs. 1D and 1E). These outcomes suggested the involvement of LSM12 in CRC pathology.

### LSM12 involved the proliferation, colony formation, migration, and invasion capabilities of CRC cells

**Figure 2:**
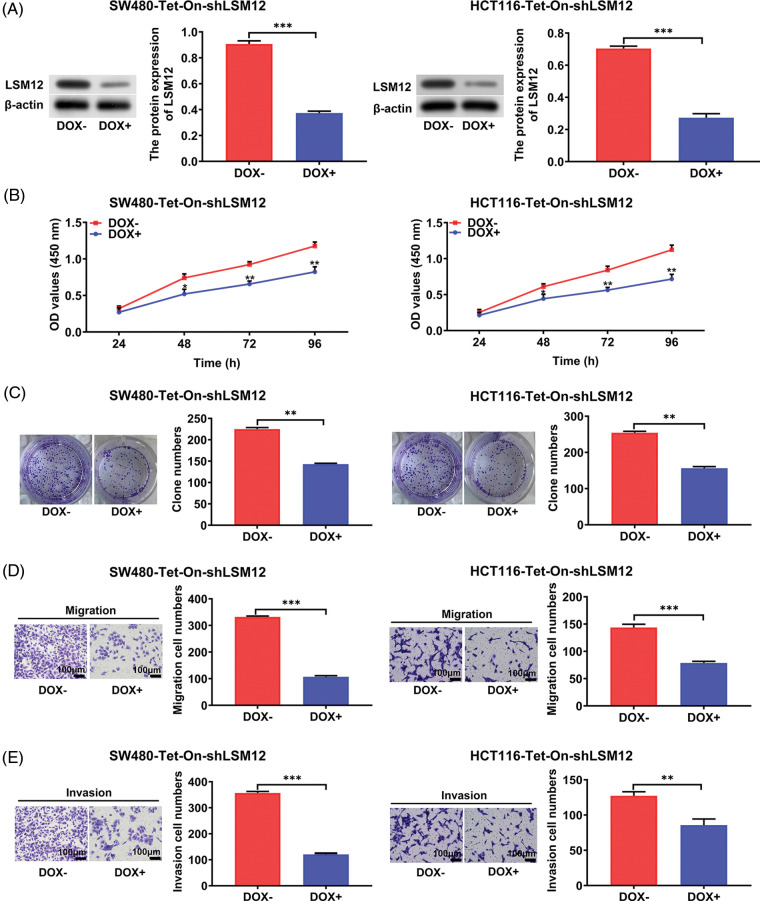
LSM12 knockdown repressed the cytopathological effects of CRC cells. (A) LSM12 knockdown was validated by Western blot in SW480-Tet-On-shLSM12 and HCT116-Tet-On-shLSM12 cells with or without DOX (Doxycycline, 100 ng/mL) treatment. The lower graph indicates the quantified LSM12 protein levels. (B) The effect of cell growth by LSM12 knockdown in SW480 and HCT116 cells was detected by CCK-8 assay at different time points. (C) The effect of cell division by LSM12 knockdown in SW480 and HCT116 cells was determined by colony formation assay. (D) The effect of cell migration by LSM12 knockdown in SW480 and HCT116 cells was assessed in the transwells. (E) The effect of cell invasion by LSM12 knockdown in SW480 and HCT116 cells was assessed in the matrigel-coated transwells. All experiments were independently conducted in triplicate technical repeats. Data shown are means ± SEM. *p < 0.05, **p < 0.01 or ***p < 0.001.

**Supplementary Figure 1:**
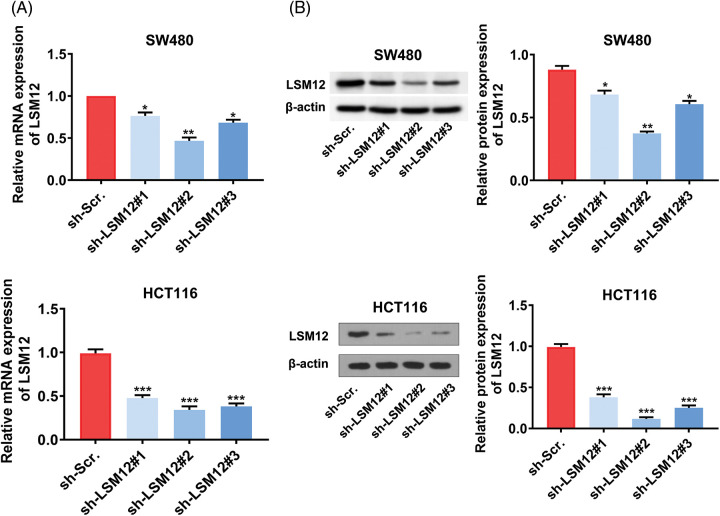
Selection of an effective LSM12 mRNA interference sequencing. (A) LSM12 mRNA expression in LSM12 knockdown (by direct shRNA system) or control SW480 cells and HCT116 cells were detected by qRT-PCR. (B) LSM12 protein expression in LSM12 knockdown (by direct shRNA system) or control SW480 cells and HCT116 cells were validated by Western blot. All experiments were independently conducted in triplicate technical repeats. Data shown are means ± SEM. *p < 0.05, **p < 0.01, or ***p < 0.001.

To understand the biological function of LSM12, we selected an effective shRNA sequencing (sh-LSM12#2) for the knockdown of LSM12 (Suppl. Fig. 1), and constructed Tet-On-shLSM12 systems in SW480 and HCT116 cells based on this sequencing (Fig. 2A). We found that the knockdown of LSM12 by DOX (Doxycycline) significantly reduced cell growth in the two CRC cell lines (Fig. 2B, *p* < 0.05) and exhibited the lower ability to divide in colony formation (Fig. 2C, *p* < 0.01). In contrast to the important role of WNT signaling in promoting tumor cell migration and invasion in CRC [10,11], knockdown of LSM12 not only effectively decreased cell migration (Fig. 2D, *p* < 0.001), but also strongly reduced cell invasion in the two CRC cell lines (Fig. 2E, *p* < 0.01 or *p* < 0.001). These results indicate LSM12, like the WNT signaling pathway, is involved in the cytopathological effects of CRC, such as cell proliferation, colony formation, migration, and invasion.

### LSM12 activated WNT signaling through stabilizing CTNNB1/LEF/TCF complex formation

**Figure 3:**
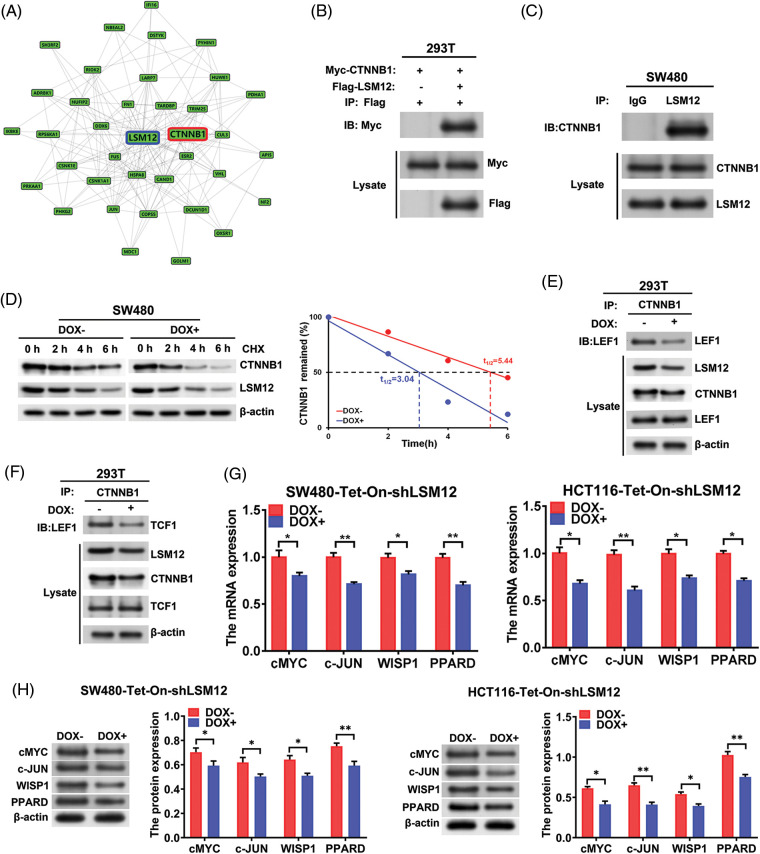
LSM12/CTNNB1 interaction activated WNT signaling. (A) Protein-Protein-Interaction (PPI) network construction of interacting proteins with LSM12 using GPS-Prot platform. (B) Interaction of LSM12 with CTNNB1 was confirmed by co-IP in Flag-LSM12 and Myc-CTNNB1 overexpressed 293T cells. (C) Endogenous interaction between LSM12 and CTNNB1 was validated by co-IP using LSM12 antibody in SW480 cells. (D) CTNNB1 protein degradation was detected by Western blot in SW480-Tet-On-shLSM12 and HCT116-Tet-On-shLSM12 cells with or without DOX (Doxycycline, 100 ng/mL) treatment following exposure to Cycloheximide (CHX,50 μg/mL) for the indicated times. The right graph indicates CTNNB1 protein degradation pattern and half-life time (t1/2). (E) Effect of LSM12 knockdown on the interaction between CTNNB1 and LEF1 was determined by co-IP in 293T-Tet-On-shLSM12 cells. (F) Effect of LSM12 knockdown on the interaction between CTNNB1 and TCF1 was validated by co-IP in 293T-Tet-On-shLSM12 cells. (G) Expression changes of WNT downstream mRNAs (cMYC, c-JUN, WISP1, PPARD) by LSM12 knockdown in SW480 and HCT116 cells were assessed by qPCR. (H) Expression changes of WNT downstream proteins (cMYC, c-JUN, WISP1, PPARD) by LSM12 knockdown in SW480 and HCT116 cells were confirmed by Western blot. All experiments were independently conducted in triplicate technical repeats. Data shown are means ± SEM. *p < 0.05 or **p < 0.01.

To evaluate the molecular mechanisms underlying the effect of LSM12 on CRC cytopathology, we simulated the LSM12 interacting proteins using the GPS-Prot Interaction Network program (http://gpsprot.org). We found that CTNNB1 (a canonical WNT signaling main transcriptional component) may interact with LSM12 (Fig. 3A). Next, exogenous co-IP was implemented to detect the interaction between Flag-LSM12 and Myc-CTNNB1 in 293T cells. As manifested in Fig. 3B, Western blot results from co-IP showed that Myc-tagged CTNNB1 protein could be immunoprecipitated interaction between LSM12 and CTNNB1. As a result, CTNNB1 protein was immunoprecipitated by an anti-LSM12 antibody rather than an anti-IgG antibody (Fig. 3C). To assess whether LSM12 interaction induced CTNNB1 protein stabilization, SW480-Tet-On-shLSM12 cells were treated with DOX and Cycloheximide (CHX, a translation inhibitor). It was found that LSM12 knockdown by DOX treatment markedly decreased CTNNB1 protein stability (Fig. 3D). Furthermore, endogenous co-IP with CTNNB1 antibody exhibited that, LSM12 knockdown repressed the binding of CTNNB1 to LEF1 and TCF1 (Figs. 3E and 3F). Thereby, all these data indicated that LSM12 knockdown suppressed a canonical WNT signaling transcriptional regulatory complex formation by inhibiting the stability of CTNNB1 protein. To further appraise LSM12-mediated WNT signaling pathway activity, WNT signaling target expression was determined in SW480-Tet-On-shLSM12 and HCT116-Tet-On-shLSM12 cells with/without DOX treatment. The results exhibited that both mRNA and protein of WNT signaling target-cMYC, c-JUN, WISP1 and PPARD were significantly repressed by LSM12 knockdown by DOX (Figs. 3G and 3H, *p* < 0.05 or *p* < 0.01). These results indicate that LSM12 involves canonical WNT signaling activation.

### LSM12 knockdown exacerbated the apoptosis of CRC cells via inhibiting CTNNB1/TCF-mediated transcription

**Figure 4:**
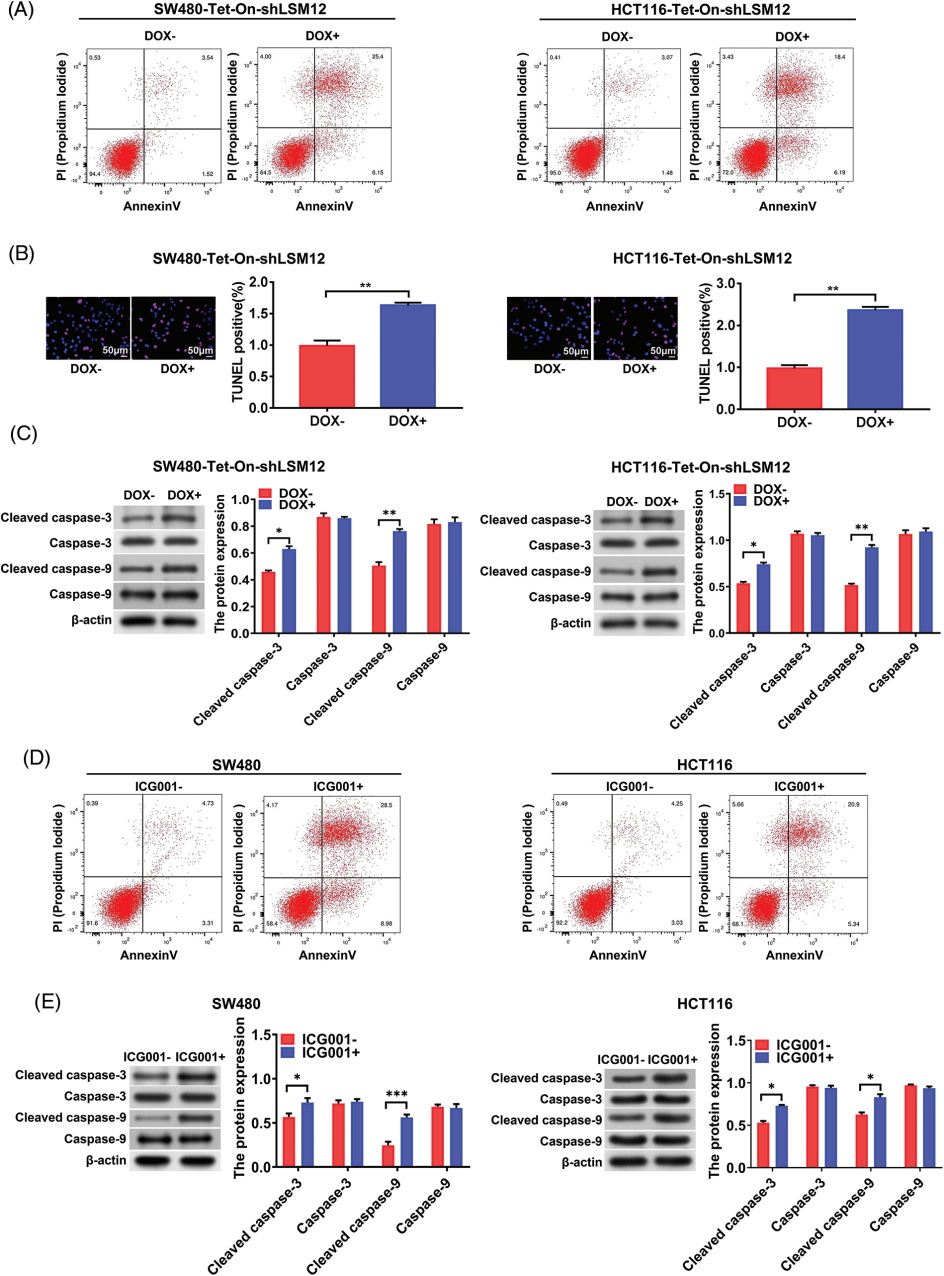
LSM12-mediated CTNNB1/TCF transcription is involved in the apoptosis of CRC cells. (A) Cell apoptosis by LSM12 knockdown in SW480 and HCT116 cells was determined by flow cytometry using Annexin V antibody and PI. (B) Cell apoptosis by LSM12 knockdown in SW480 and HCT116 cells was confirmed by TUNEL assay. The right graph indicates the quantification of TENEL-positive cells. (C) The cleaved levels of Caspase-3 and -9 by LSM12 knockdown in CRC cells were assessed by Western blot. (D) Cell apoptosis by ICG001 (10 μM) treatment in SW480 and HCT116 cells was determined by flow cytometry using Annexin V antibody and PI. (E) The cleaved levels of Caspase-3 and -9 by ICG001 treatment in CRC cells was assessed by Western blot. All experiments were independently conducted in triplicate technical repeats. Data shown are means ± SEM. *p < 0.05, **p < 0.01 or ***p < 0.001.

Although it has been reported that the WNT signaling pathway makes CRC cell chemoresistance by inhibiting apoptosis [12], whether the inhibition of transcriptional regulation of CTNNB1/LEF/TCF complex directly leads to CRC cell apoptosis remains unclear. However, when we constructed LSM12 knockdown CRC cells by shRNA (Suppl. Fig. 1), we found that stable cells could not be successfully selected due to cell death. So we used the Tet-On system to conditionally knockdown LSM12 in CRC cells for the following experiments. Based on these results, we hypothesized that the LSM12 and its associated CTNNB1/LEF/TCF transcriptional mechanism might be involved in the apoptosis of CRC cells. For this, we first determined the CRC cell apoptosis by LSM12 conditional knockdown in SW480-Tet-On-shLSM12 or HC116-Tet-On-shLSM12 cells. We found Annexin V and PI dual positive apoptotic cells was increased by 7 or 6 folds by LSM12 knockdown, respectively (Fig. 4A). Furthermore, LSM12 knockdown in both CRC cells markedly increased TUNNEL positive apoptotic cell population (Fig. 4B, *p* < 0.01). Associated with these cellular events, an apoptotic molecular mechanism such as Caspase 3 and Caspase 9 cleavages was significantly activated by LSM12 knockdown in both CRC cells (Fig. 4C, *p* < 0.01). To assess whether the LSM12-mediated CTNNB1/TCF transcriptional mechanism involves CRC cell apoptosis, we treated CTNNB1/TCF transcriptional inhibitor ICG001 to CRC cells. Similar to LSM12 knockdown, ICG001 treatment not only increased cell apoptosis (Fig. 4D), but also induced Caspase 3 and Caspase 9 cleavages in SW480 and HCT116 cells (Fig. 4E, *p* < 0.05 or *p* < 0.001). These results suggested that blocking LSM12-CTNNB1/TCF axis directly induces CRC cell apoptosis, maybe an excellent therapeutic target point for CRC.

### LSM12 knockdown repressed tumorigenicity of CRC cells

**Figure 5:**
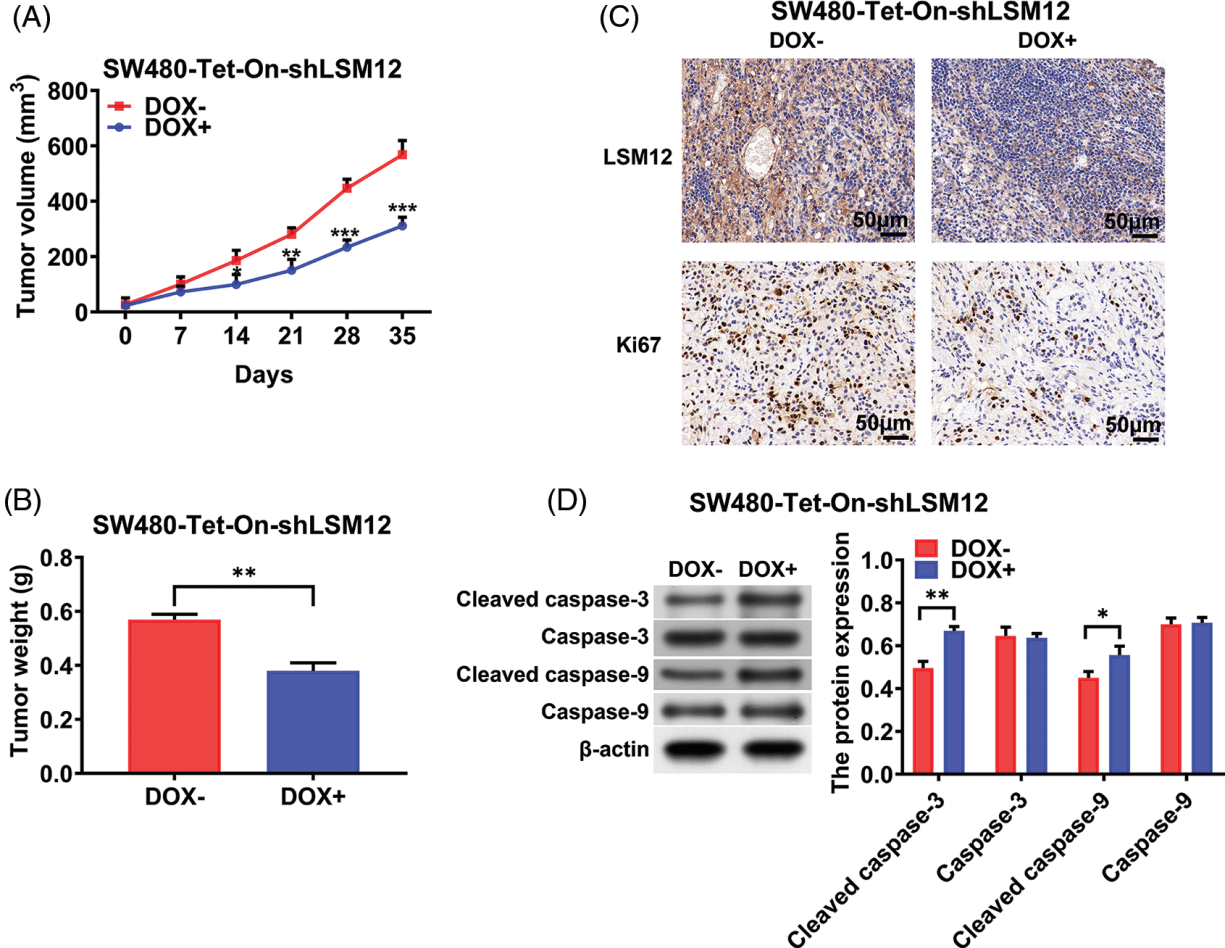
LSM12 knockdown repressed the growth of CRC xenograft tumor. (A) The effect of LSM12 knockdown in SW480-Tet-On-shLSM12 cells on the xenograft tumor growth was monitored with the tumor volume under indicating time points. (B) The tumor weights at the end time point (35 days) was compared between LSM12 knockdown and control tumors. (C) LSM12 knockdown and Ki67 positive cell population were observed by immunohistochemical staining on xenograft tumor tissues. (D) The cleaved levels of Caspase-3 and -9 by LSM12 knockdown in xenograft tumors were assessed by Western blot. All experiments were independently conducted in triplicate technical repeats. Data shown are means ± SEM. *p < 0.05, **p < 0.01 or ***p < 0.001.

To further understand the tumorigenic role of LSM12, SW480-Tet-On-shLSM12 cells were subcutaneously injected into nude mice, and induced LSM12 knockdown by DOX from day 7 after injection. As shown in Figs. 5A and 5B, LSM12 knockdown significantly reduced tumor growth and weight (*p* < 0.05, *p* < 0.01 or *p* < 0.001). These tumor repressive effects of LSM12 knockdown were associated with reducing Ki67 positive proliferating cell population (Fig. 5C), and increasing apoptotic marker cleaved caspase-3/-9 levels (Fig. 5D, *p* < 0.05 or *p* < 0.01). Taken together, these results implied that LSM12 and its downstream WNT signaling-associated cellular functions, such as cell proliferation and apoptosis, play an important role in CRC tumorigenesis.

## Discussion

LSM12 possesses multiple functions, such as regulating mRNA degradation and DNA replication stress [13,14]. It is one of the main factors participating in DNA damage induced by oxidative stress [15]. Heretofore, the research on the function of LSM12 in human malignant tumors is still very limited [16–18]. Here, we demonstrated that LSM12 is involved in cytopathologic and tumorigenic functions during CRC development through the regulation of CTNNB1 protein stability and CTTNB1-LEF1-TCF1 transcriptional complex formation. This article not only accurately demonstrated the function of LSM12 in the progression of CRC, but also proposed a new molecular mechanism as the upstream of the WNT signaling pathway in CRC tumorigenicity.

As a critical mutation pathway of CRC, the WNT signaling pathway is considered the most essential molecular targeting signaling pathway for CRC therapy. At present, the main therapeutic strategies for targeting WNT signaling in CRC are focused on inhibiting CTNNB1 expression and protein accumulation, because 75% of CRC patients have APC mutation [2,3]. In fact, the pathological function of WNT signaling is dependent on CTNNB1 nuclear translocation to perform transcriptional regulator, so the relevant mechanism is a hot issue for understanding pathologic WNT signaling in CRC [19–22]. In this study, we found that LSM12 directly interacts with CTNNB1 to regulate the formation of the CTNNB1-LEF1-TCF1 transcriptional complex. Actually, LSM12 also plays ion and protein transfer functions on the membrane of endolysosome and nucleus [5,23], indicating it possibly involved in CTNNB1 nuclear translocation. On the other hand, whether the binding of LSM12 with CTNNB1 is maintained into the nucleus and involved in the intentional transcription of WNT signaling downstream genes such as cMYC, c-JUN, WISP1 and PPARD remains to be further investigated.

Although the accumulated evidence indicate that CTNNB1-LEF1-TCF1 transcriptional target genes, such as MDR1 (multidrug resistance protein 1), Survivin, and MMP7 (matrix Metallopeptidase 7), act as chemoresistance factors in CRC [12,24–26], it is unclear whether inhibition of WNT signaling alone can achieve the therapeutic effect of CRC. However, other evidence suggest that AXIN1 (axis inhibition proteins 1) deubiquitin ligase USP44 (ubiquitin-specific peptidase 44) and LRP6 (low-density lipoprotein receptor-related protein 6) antagonist VSTM2A (V-set and transmembrane domain containing 2A) induce apoptosis of CRC cells and inhibit the growth of CRC by inactivating WNT signaling [27,28]. Also, we found LSM12 knockdown and ICG001 treatment enhanced the apoptosis of CRC cells, implying the possibility of direct inhibition of WNT signaling to achieve a therapeutic effect in CRC.

In the present work, LSM12 was demonstrated to play a tumorigenic function in CRC by regulating WNT signaling via interaction with CTNNB1 to activate CTNNB1-LEF1-TCF1 complex transcription. Direct targeting LSM12-CTNNB1 transcription could achieve CRC cellular apoptosis, implying LSM12 might be a promising target for CRC treatment. Drugs with inhibitory function against LSM12 will help provide new options for the treatment of CRC patients. Of course, more efforts still need to be made in the future.

## Data Availability

The analyzed data sets generated during the study are available from the corresponding authors upon reasonable request.
